# Isolation, separation, identification, and quantification of bioactive methylated flavone regioisomers by UHPLC‐MS/MS

**DOI:** 10.1002/ansa.202100016

**Published:** 2021-05-05

**Authors:** Hafiza Noreen, Edward N. Smith, Muhammad Farman, Tim D.W. Claridge, James S.O. McCullagh

**Affiliations:** ^1^ Department of Chemistry Quaid‐i‐Azam University Islamabad Pakistan; ^2^ Department of Chemistry Government College Women University Faisalabad Pakistan; ^3^ Chemistry Research Laboratory Department of Chemistry University of Oxford Mansfield Road Oxford UK

**Keywords:** *Coronopus didymus*, flavonoids, methylated flavone, product ions, regioisomers

## Abstract

Methylated flavones, commonly found in many plants of the Brassicaceae family, have potent antioxidant and anticancer activity with diverse therapeutic potential. However, the specific regioisomers of methylated flavones can have significantly different biochemical and potentially therapeutic properties as shown by various bioassays but analytically differentiating these compounds has been technically challenging and rarely reported. In this study, we demonstrate differentiation and identification of selected bioactive methylated flavone regioisomers, namely 5,7,3′‐trihydroxy‐4′‐methoxyflavone, and 5,7,4′‐trihydroxy‐3′‐methoxyflavone extracted from *Coronopus didymus*, a member of the Brassicaceae family, using ultra‐performance liquid chromatography coupled with electrospray ionization quadrupole time‐of‐flight tandem mass spectrometry (UPLC‐ESI‐QTOF‐MS/MS). Characteristic MS/MS product ions produced from neutral loss of carbon monoxide, and a methyl radical from the [M‐H]^–^ ion, exhibited differential relative abundances attributed to different structural stabilities under the same activation and collision‐induced dissociation conditions. MS/MS also provided structural information which was sufficient to differentiate the methylated regioisomers and determine the position of the methyl group based on interpretation of their respective fragmentation patterns. Quantification showed 5,7,4′‐trihydroxy‐3′‐methoxyflavone was at least 1.60 mg per 10 g plant material in *C. didymus* extracts. This study demonstrates a straightforward and novel approach to rapidly differentiate, identify and quantify regio‐isomeric methylated flavone natural products using reversed‐phase UPLC‐MS/MS.

**ABBREVIATIONS**
*C. didymus*
: *coronopus didymus*
CID: collision‐induced dissociation
Da: dalton
LLOD: lower limit of detection
LLOQ: lower limit of quantification
UHPLC‐MS/MS: ultra‐high performance liquid chromatography‐tandem mass spectrometry

## 1 INTRODUCTION

Flavonoids constitute a very large and important group of polyphenolic natural products present in almost all plant species. Approximately 13,000 different flavonoid structures have been reported to date,^1^ including a wide range of regioisomeric methylated flavones^2^, ^3^ which exhibit important biological and therapeutic properties. For example, diosmetin (5,7,3′‐trihydroxy‐4′‐methoxyflavone) has been shown to possess chemo‐preventive, anti‐mutagenic, anti‐allergic, anti‐osteoporosis,^4^ anti‐pigmentation,^5^ iron‐chelating, and antioxidant activity.^6^ Diosmetin has been used for the treatment of cerulein‐induced acute pancreatitis in mice.^7^ The diosmetin and luteolin have been shown to exert synergistic cytostatic effects in human hepatoma (HepG2) cells.^8^

The regioisomer, chrysoeriol (5,7,4′‐trihydroxy‐3′‐methoxyflavone) possesses antioxidant and anti‐inflammatory,^9^ antitumor,^10^ antimicrobial, and selective bronchodilator effect.^11^ It potentially serves as a novel cardioprotective agent against doxorubicin‐induced cardiotoxicity.^12^ Chrysoeriol has shown antitumor activity against human multiple myeloma cells in vitro^13^ and modulates the downstream signal transduction pathways of platelet‐derived growth factor, for the treatment of vascular diseases and protects osteoblasts from oxidative stress‐induced toxicity.^14^, ^15^

In previous work, we reported that the regioisomeric methylated flavone (5,7,4′‐trihydroxy‐3′‐methoxyflavone) with 3′‐OCH_3_, isolated from *Coronopus didymus* Linn. (lesser swinecress), had 45 times more potent cytotoxic activity against HeLa cells and 9 times more cytotoxic against LN18 cancer cells, compared to its regioisomer (5,7,3′‐trihydroxy‐4′‐methoxyflavone) with 4′‐OCH_3_.^16^ *C. didymus* is enriched in a large number of volatile phytochemicals and methylated flavone regioisomers with high antioxidant and cytotoxic activity.^16^, ^17^

In order to provide a better understanding of therapeutic potential, it is important to distinguish and identify regio‐isomeric methylated flavones from natural sources, however, their structural characterization is a challenging task, in part due to a general lack of authentic standards. Mass spectrometry (MS) can differentiate compounds by *m/z*, but chromatographic separation and tandem approaches (MS/MS) are required to differentiate regioisomers that share the same *m/z* value.^18^ However, the extent to which these techniques can be successfully applied in combination to determine regio‐specificity of methylated flavone natural products has been little explored to date.

Given the potent cytotoxic activity of the methylated flavone and therefore the therapeutic potential of the plant, we designed and implemented a protocol to extract, separate, identify and quantify methylated regioisomers in extracts from the aerial parts of *C. didymus*, a member of the Brassicaceae family. We used UPLC‐MS/MS to differentiate 5,7,4′‐trihydroxy‐3′‐methoxyflavone (**1**) and 5,7,3′‐trihydroxy‐4′‐methoxyflavone (**2**) which, to the authors' knowledge, is the first differentiation of regio‐isomeric methylated flavones with methylation at alternate positions. This study demonstrates the selectivity and sensitivity of an LC‐MS/MS approach for investigating regioisomers of methylated flavones in plant extracts and provides a tool for future studies investigating the discovery and therapeutic potential of this important class of natural products.

## 2 MATERIAL AND METHODS

### 2.1 Chemicals and instruments

All HPLC‐grade solvents and 5,7,3′‐trihydroxy‐4′‐methoxyflavone were purchased from Sigma‐Aldrich (Gillingham, UK). Extract and fractions were evaporated in vacuo using a rotary evaporator (BÜCHI Rotavapor R‐200, Switzerland). Pre‐coated Silica gel 60 F_254_ plates (0.25 mm, Merck) were used for thin‐layer chromatography (TLC). Sephadex LH‐20 (Sigma Life Science, Sweden) was used for size exclusion chromatography (SEC). UV‐Vis. spectra were recorded on UV‐Vis. Spectrophotometer (UV‐1700 PHARMASPEC, Shimadzu, Japan) and data were processed by UVProbe Version 2.00. Waters Xevo G2‐S Q‐TOF mass spectrometer equipped with a Waters Acquity UPLC system (Waters, Elstree, UK) was used for UPLC‐MS and MS/MS analyses. A Bruker AVII 500 MHz Nuclear Magnetic Resonance (NMR) spectrometer was used for ^1^H‐NMR.

### 2.2 Extraction and purification

Aerial parts of plant *C. didymus* were collected in spring 2015 from Sector I‐8/1 Islamabad, Pakistan. The ethanol extract was prepared by soaking dried aerial parts of *C. didymus* (10 g) for 96 h in extractant (100 mL). The extracts were ultrasonicated for 30 min., filtered, and evaporated to dryness under reduced pressure at 45°C to get 2.0 g crude ethanolic extract of *C. didymus* aerial parts (CDA Et). The phenolic extract of *C. didymus* aerial parts (CDA MW) was prepared by dissolving the ethanolic extract (2.0 g) in methanol‐water in a ratio of 7:3 (v/v), to get 0.67 g of extract CDA MW, which was purified by SEC using Sephadex LH‐20 (90 cm×2.2 cm) column using the same eluent, at a flow rate of 1 mL/min to furnish 10 fractions (F1‐F10). All the fractions were analyzed by combined TLC analysis using pre‐coated Silica gel plates. One pure fraction F10 containing a high amount of Compound **1** was obtained as determined from its TLC analysis. After concentration, Compound **1** (1 mg) was obtained as a yellow precipitate from pure fraction F10. Compound **1** in the fraction F10 was analyzed by UV‐Vis. and NMR Spectroscopy, UPLC‐MS, and UPLC‐MS/MS.

### 2.3 UPLC‐ESI‐MS and MS/MS analyses

UPLC‐MS and MS/MS analyses were performed using the Waters Xevo G2‐S Q‐TOF mass spectrometer equipped with a Waters Acquity UPLC system (Waters, Elstree, UK). Separation was achieved using a Merck Purospher^®^ STAR RP‐18 end‐capped HLPC columns (3 µm particle size), (Hibar^®^ HR 50‐2.1 mm, UHPLC Column, Merck, Germany). A 5 µL full loop injection was used for qualitative analyses. The chromatographic mobile phases were 0.1% formic acid in milli‐Q water for mobile phase A and acetonitrile for mobile phase B. The flow rate was 0.2 mL min^–1^, with a gradient elution program consisting: 0‐1.0 min isocratic 5% B, 1.0‐8.0 min linear gradient from 5‐99% B, 8.0‐10.0 min maintained for isocratic 99% B, with 10.0‐12.0 min for initial conditions of 5% B for column equilibration. The total run time was 12 min. The chromatographic eluate was directly introduced into the ESI source for MS detection in negative ionization mode.

The mass spectrum of the isolated Compound **1** from the plant fraction F10 was acquired in negative‐ion ESI mode using an *m/z* range of 60‐300 and mass resolution of approximately 30000. Negative‐ion electrospray was used with a capillary voltage of 2 kV; cone voltage, 60 V; source offset, 80 V; collision energy, 15 eV; desolvation gas temperature, 400°C; source temperature, 100°C; and desolvation gas flow, 600 L/hr. Samples were analyzed by UPLC‐MS/MS using a single ion monitoring (SIM) experiment. The precursor ion [M‐H]^–^ corresponding to the deprotonated form of Compound (**1**) at *m/z* 299 was selected by the quadrupole mass analyzer (MS^1^) transmission window and subsequently subjected to collision‐induced dissociation (CID) fragmentation and TOF mass spectral analysis. Experimentation with 20, 30, and 40 eV collision energies were optimized to obtain optimal MS/MS spectra of Compound **1**. A collision energy ramp from 20‐40 eV was then chosen and product ions were analyzed by MS/MS scanning in the 100‐300 *Da* range. The MS/MS parameters were as follows; AGC target, 1e5; Max IT, 50 ms; Loop count, 5; isolation window, 1.0 *m/z*; and intensity threshold, 2e4. Chromatographic data were collected and processed by using MassLynx software (Micromass, Altringham, UK).

A 20‐40 eV collision energy was chosen by initially analyzing Compound **1** in fraction F10 by 20, 30, and 40 eV collision energy. It was found that at lower energy (20 eV), two product ions were obtained i.e., *m/z* 284.1743 and 256.1845. At 30 eV collision energy, five product ions at *m/z* 284.1743, 256.1845, 227.1854, 151.1565, and 107.1619 were obtained. At 40 eV, 10 main product ions were formed at *m/z* 284.1743, 256.1845, 227.1854, 211.1909, 199.1928, 183.1994, 151.1565, 133.1817, 107.1619 and 83.1549 (Supplementary information, Figure N1).

### 2.4 Quantitative analysis

Relative quantitation of Compound **1** in the fraction F10 from the ethanolic extract was performed using a calibration curve constructed with an authentic standard of 5,7,3′‐trihydroxy‐4′‐methoxyflavone (Compound **2**). A stock solution of the Compound **2** was prepared in methanol at a concentration of 1 mg/mL and diluted in methanol to five different concentrations; 5, 15, 25, 50, and 100 µg/mL. These samples were analyzed in triplicate on the same UPLC system (see Section 2.3). A calibration curve (Supplementary information, Figure N2) was constructed between different concentrations and the peak area, obtained from HPLC analysis of different concentrations of standard and to determine a linear response over the concentration range, validity of data, lower limit of detection (LLOD) and lower limit of quantification (LLOQ). The fraction F10 (100 µL/mL) with an unknown concentration of Compound **1** obtained from *C. didymus* (see Section 2.2) was run after the standard on the UPLC‐MS with a Waters Xevo G2‐S Q‐TOF system under the same solvent condition as above (see Section 2.3) to obtain a peak area. LLOD and LLOQ were calculated by the standard deviation (σ) of y‐intercept of the regression line and the slope (S) of the regression line using equations for the calibration method as; LLOD = 3×σ/S and LLOQ = 10×σ/S. The calibration curve obtained using the standard was then used to calculate the concentration of Compound **1** in the fraction F10 of the ethanolic extract.

## 3 RESULTS AND DISCUSSION

**FIGURE 1:**
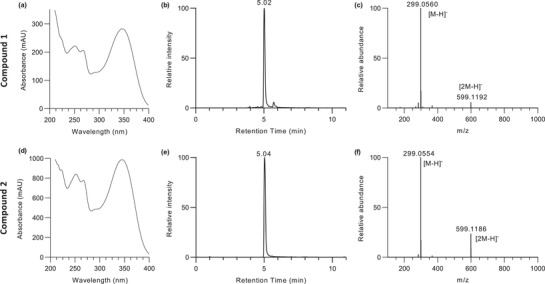
UV‐Vis. spectra, TIC profile, and mass spectra of 5,7,4′‐trihydroxy‐3′‐methoxyflavone (Compound **1**) in the fraction F10 of *C*. *didymus* and standard 5,7,3′‐trihydroxy‐4′‐methoxyflavone (Compound **2**)

Wild *C. didymus* was collected from its natural habitat around the city of Islamabad in Pakistan and the aerial parts were ethanol extracted and subjected to size‐exclusion chromatography followed by TLC and UV‐Vis. Spectroscopy (see Section 2.2). Analysis of fraction (F10) revealed the presence of a single major compound and UV‐Vis. absorption spectra (Figure 1a) suggested flavone or flavonol subgroups.^19^

### 3.1 UPLC‐ESI‐MS analyses

UPLC‐MS analysis of fraction F10 from the ethanolic extract of *C. didymus* was performed. A major chromatographic peak was obtained on the TIC at a retention time of t_R_ = 5.02 min. (Figure 1b). MS spectra were collected in negative ion mode because this provided the highest ion intensity. The base peak in the mass spectrum was obtained at t_R_ = 5.02 min. with an *m/z* value of 299.0560, consistent with the chemical formula C_16_H_12_O_6_ with 1.34 ppm error from the theoretical *m/z* value for the chemical composition of Compound **1** (Figure 1c). Hereafter the compound corresponding to this *m/z* value is referred to as “Compound **1″**. In the mass spectra (Figure 1c) it was observed that the peak at the highest *m/z* value of 599.1192 was not the deprotonated molecule [M‐H]^–^ but rather the molecular complex [2 M‐H]^–^.

In addition to accurate mass enabling chemical formula prediction, additional confirmation that the *m/z* 299.0560 and 599.1192 peak corresponded to the [M‐H]^–^ and dimer respectively was given by the isotope abundance patterns which showed the same peak abundance profile. Their respective accurate mass values predicted the deprotonated monomer and dimer of “Compound 1″ to within 5 ppm. The *m/z* corresponding to the deprotonated form of Compound **1** was 299, 14 *Da* higher than tetrahydroxyflavone (*m/z* = 285). This was consistent with Compound 1 bring monomethylated tetrahydroxyflavone. An authentic standard of the monomethylated tetrahydroxyflavone, 5,7,3′‐trihydroxy‐4′‐methoxyflavone was purchased (hereafter referred to as Compound **2**) and analyzed by UV‐Vis. and UPLC‐MS for comparison with Compound **1**.

The UV‐Vis. spectrum of Compound **2** in the region 200‐400 nm is shown in Figure 1d. Compound **2** provided two major absorption peaks one at 216inflection, 222, 252, 268 nm with a shoulder at 290 nm (Band II) and other at 346 nm (Band I). As shown in Figure 1d, the maximum absorption of Band I in the MeOH spectra appeared at 346 nm for Compound **2**, which is characteristic of the flavone subgroup.^19^ From the UV spectra it was observed that the band II at 251 nm was broad (Figure 1a) for Compound **1** while it was sharp at 252 nm (Figure 1d) for Compound **2**. The presence of a Band II at 222 nm for Compound **2** and its absence in Compound **1** helped to address the minor difference in the UV pattern of these two flavones.

UPLC‐MS analysis of the authentic standard (Compound **2**) provided retention time, *m/z*, and accurate mass measurements. Compound **2** had a TIC chromatogram retention time at t_R_ = 5.04 min. (Figure 1e). Based on previous reports using reverse phase chromatography, the effects of methylating single hydroxyl groups on lengthening retention of flavone analogs are 7 > 3′ (5′) ≈4′ > 3 > 5 number carbon.^20^, ^21^, ^22^ Using this as a guide to retention time differences, the retention time of Compound **1** was compared with the standard flavone (Compound **2**). Supplementary information (Figures N3 and 4) shows a comparison of the retention times. Based on the relatively close retention time with Compound **1**, it was concluded that the B‐ring substituent was likely to be methylated at the 4′ or 3′ (5′) positions but this needed to be investigated further.

The ESI‐mass spectra of Compound **2** (*t*_R_ = 5.04) in negative ion mode (Figure 1f) showed a deprotonated molecule [M‐H]^–^ at an *m/z* value of 299.0554, which was consistent with the chemical formula C_16_H_12_O_6_ with ‐0.70 ppm mass accuracy (Figure 1f). The peak at *m/z* 599.1186 was attributed to the dimer, i.e., [2 M‐H]^–^ and similarly to the LC‐MS analysis of Compound **1** was attributed to dimerization resulting from the ionization process.

**FIGURE 2:**
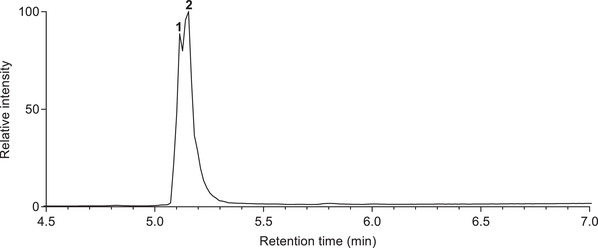
TIC profile of the spiked sample of Compound **1** and **2**

UPLC‐MS/MS analysis of the authentic standard (Compound **2**) in triplicate at five different concentrations; 5, 15, 25, 50 and 100 µg/mL was performed and the results compared with a similar analysis of the fraction F10 containing the single peak (labeled Compound **1**). TICs for deprotonated molecules of Compound **1** and **2** at 5 different concentrations are provided in Supplementary information (Figures N3 and 4). Although the retention times for the two analytes were very close (they differed by 0.02 min. (1.2 sec)) this difference was consistently reproducible over the replicate analyses (Supplementary information, Figures N3 and 4). This suggested two different compounds were present with the same chemical formula that were eluting 0.02 min apart. Next, fraction F10 and the authentic standard labeled Compound 2 were combined and made up to a concentration of 100 µg/mL and then analyzed by UPLC‐MS. As the difference of retention time between the two compounds was very small two overlapping peaks with the apex at t*_R_* 5.02 and 5.04. min were observed (Figure 2).

### 3.2 UPLC‐MS/MS analyses

**FIGURE 3:**
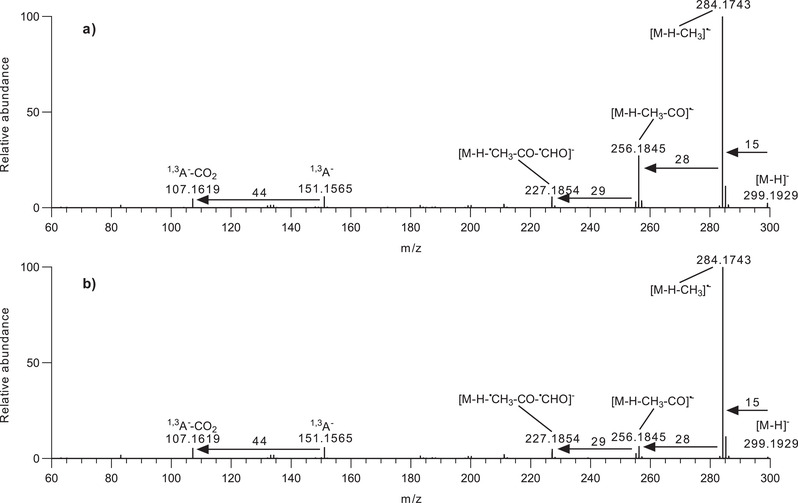
The diagnostic tandem mass spectrometry (MS/MS) product ion spectra for the [M‐H]^–^ at *m/z* 299 for (**a**) Compound **1** and (**b**) Compound **2**

**SCHEME 1:**
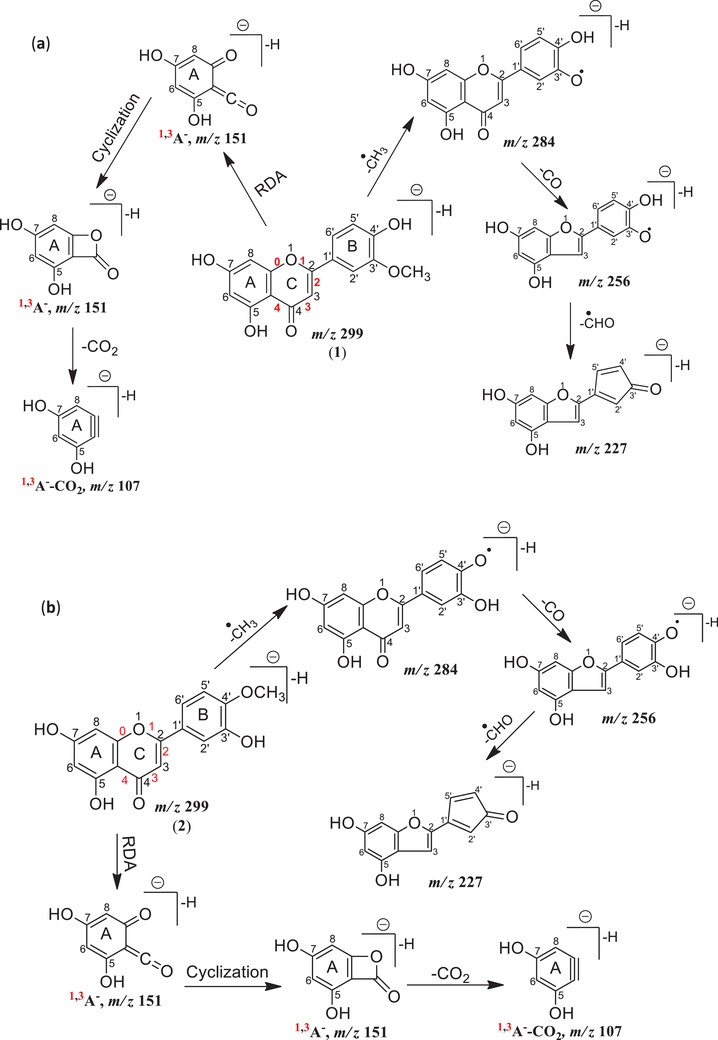
Proposed CID fragmentation mechanism for deprotonated molecules of (**a**) Compound **1** and (**b**) Compound **2**

Next structural differences were explored between the two compounds using MS/MS. Samples containing F10 and the authentic (Compound **2**) were analyzed separately using UPLC‐ESI‐MS/MS with collision‐induced dissociation (see Section 2.3). Figures 3a and b shows the fragmentation spectra from MS/MS analysis (isolation window, 1.0 *m/z*) using *m/z* 299.1929 as the precursor at t_R_ = 5.02 min. and t_R_ = 5.04 min. respectively. Similar product ions are observed at both retention times but the relative abundance of the precursor ion peak [M‐H]^–^ was 3.53 in Fraction F10 while it is 1.09 for a standard solution of Compound **2** (Figure 3). Both sets of fragmentation spectra were analyzed and interpreted based on the differences in product ion spectra and accurate mass analysis of product ions (Scheme 1).

UPLC‐MS/MS analyses of samples containing Compound **1** and **2** were performed in triplicate using the same collision energy ramp from 20‐40 eV, at five different concentrations; 5, 15, 25, 50, and 100 µg/mL (Supplementary information, Figures N5 and 6). At these different concentrations the mass spectral fragments and their relative abundances, for both Compound **1** and **2**, were reproducible (Supplementary information, Figures N5 and 6).

A comparison of the product ion spectra using the same collision energy ramp (20‐40 eV) for Compound **1** (Figure 3a), reveals a very similar series of peaks to the product ion spectrum for Compound **2** (Figure 3b). However, there are some differences between the two spectra, particularly in terms of product ion abundance. It should be noted that product ion abundances from CID can differ between different instruments, or even for the same instrument over time, due to differences in the CID gas pressure and transmission voltages but for sequential analysis of samples on the same instrument, highly reproducible abundances can be collected. Some of the CID product ions established by accurate mass are illustrated in Scheme 1. The production ion spectra were interpreted as follows: The base peak product ion at *m/z* 284.1743 (100%) was observed from the CID of the deprotonated flavone (Compounds **1** and **2**) and corresponded to the loss of a methyl radical (‐15.0190 *Da*) from the deprotonated molecule resulting in an [M‐H‐CH_3_]^•–^ radical anion, which confirmed that both the compounds were monomethylated flavones. No further loss of a methyl group was observed in the product ions. As Li et al. reported, neutral loss of CH_4_ is diagnostic for myricetin derivatives, that have two or more *O*‐methyl groups on the B‐ring, provided that no methyl group is present on the 3 position.^23^ Justesen demonstrated homolytic cleavage as the mechanism for loss of *^•^* CH_3_.^24^ Phenolic methyl ether groups show this characteristic ion transition from even to odd electron. The [M‐H‐CH_3_]^•–^ ions of *m/z* 284.1743 are predominant fragments for both Compounds (**1** and **2**), owing to the formation of a relatively stable anion structure. The methyl groups on the flavones are cleaved as a methyl radical in a position‐independent manner. This interpretation is speculation and is suggested as it logically fits the mass changes upon fragmentation, however, proof of its structural correctness was not pursued using isotope studies or computational simulations as this was not the main aim of the study which was to demonstrate that UPLC‐MS could be used to separate and identify the methylated isomers.

The methoxylated flavones also exhibit a product ion at *m/z* 256.1845 corresponding to concurrent loss of a methyl radical (‐15.0190 *Da*) and neutral CO (‐27.9898 *Da*) to give a fragment [M‐H‐CH_3_‐CO]^•–^ in both spectra (Figure 3) although at different abundances for the two compounds (Supplementary information, Table N1). The loss of a CO group could occur from any oxygenated carbon atom in both molecules. However, CO removal from the C‐ring (Schemes 1a and b) involving the C‐4 atom is theoretically favorable compared with CO removal from a benzene ring (both A and B‐rings). The *m/z* 256.1845 ion due to the methyl radical and CO loss was observed here as the second most abundant fragment in the spectra of Compound **1** with 36.16% relative abundance (Supplementary information, Table N1), but appeared as a minor fragment in the mass spectrum of Compound **2** with a relative abundance of 9.97% (Supplementary information, Figure N7).

The fragment ions at *m/z* 227.1854 (7.49%) (Supplementary information, Table N1) are consistent with the sequential loss of a methyl radical (‐15.0190 *Da*), neutral CO (‐27.9898 *Da*) and formyl radical (^∙^CHO) (‐29.0027 *Da*) [M‐H‐^∙^CH_3_‐CO‐^∙^CHO]^–^ from the deprotonated precursor ions (Scheme 1). The most useful and diagnostic fragments for flavonoid aglycone identification and structural elucidation were at *m/z* 151.1565 and 107.1619. These were formed by cleavage of carbon‐carbon bonds from the C‐ring of the flavonoids (Scheme 1) rationalized by retro‐Diels‐Alder fragmentation and named according to the nomenclature proposed by Ma *et al*.^25^ The main advantage of RDA fragmentation of flavonoids is that it provides information about the number and type of substituents present in the A‐, B‐, and C‐rings, by consolidating the extra masses added to each ring. The product ions ^1,3^A^–^ and ^1,3^A^–^‐CO_2_ at *m/z* 151.1565 and 107.1619 were found to constitute the characteristic fragments of the flavones. The product ion ^1,3^A^–^, which contains the whole A‐ring as well as the O_1_, C_4,_ and O_4_ atoms, is more abundant than ^1,3^A^–^‐CO_2_ product ion. So the RDA fragment at *m/z* 151.1565 indicated that an extra mass equals two hydroxyl groups (*m/z* 32) added to the characteristic A‐ring of flavone. This fragment also indicated that two ‐OH groups were attached to the A‐ring. The appearance of ^1,3^A^–^ and ^1,3^A^–^‐CO_2_ ions further indicated that these fragments are not methylated (Scheme 1), which indicated that methylation of C_5_ and C_7_ hydroxyls was unlikely. Therefore, both compounds have methylation on the B‐ring. It was found that retro‐Diels‐Alder fragmentation of methylated flavones can generate fragments that retained the methoxy group at the original methylated ring of the flavone.

The peak at *m/z* 151.1565 (8.50%) was interpreted as a fragment ^1,3^A^–^ (Figure 3), (intact A‐ring and superscripts 1,3 indicating C‐C bonds at 1 and 3 positions of C‐ring (Scheme 1, number in red color) have been broken. This fragment ^1,3^A^–^ on cyclization and subsequent loss of a neutral molecule of CO_2_ (‐43.9946 *Da*) provided a fragment ^1,3^A^–^‐CO_2_ at *m/z* 107.1619 with 6.12% relative abundance in Compound **1** (Supplementary information, Table N1). These two fragments at *m/z* 151.1565 and 107.1619 provided further evidence that methyl groups were present on the B‐ring in both compounds. Previously^16^ additional evidence regarding the presence of a substitution (hydroxylation) at 5 and 7 positions, was provided by ^1^H‐NMR, where the A‐ring of Compound **1** was represented by two meta‐coupled doublets at *δ*_H‐6_ 6.236 (d, ^4^ *J* = 2.1 Hz) and *δ*_H‐8_ 6.499 (d, ^4^ *J* = 2.1 Hz) with same coupling constant (Supplementary information, Table N2).

From fragments at *m/z* 151.1565 and 107.1619, it was confirmed that two hydroxyl groups were present on the A‐ring while a deprotonated molecule appeared at *m/z* 299 in both samples so it was clear that the methyl group was present on the B‐ring along with one hydroxyl group. All the major fragments for both the compounds appeared at the same *m/z* values (Scheme 1). Based on the different fragment intensities (abundances) of the major fragments it was concluded that Compound **1** and **2** had hydroxyl and methoxy groups interchanged on the B‐ring.

**FIGURE 4:**
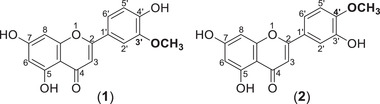
The chemical structures of 5,7,4′‐trihydroxy‐3′‐methoxyflavone (Compound **1**) isolated from *C. didymus* aerial parts and its regioisomer 5,7,3′‐trihydroxy‐4′‐methoxyflavone (Compound **2**) used as a standard in the current study

The position of the methoxy group for the Compound **2** standard was C‐4′ (5,7,3′‐trihydroxy‐4′‐methoxyflavone) while Compound **1** from fraction F10 had a methoxy group at C‐3′ (5,7,4′‐trihydroxy‐3′‐methoxyflavone) (Figure 4). Relative abundance of the product ions spectra (Supplementary information, Table N1), accurate mass analysis of precursors and products as well as additional flavonoid MS/MS fragmentation available in literature was used for structural identification.^26^, ^27^, ^28^ To the best of our knowledge this is the first report on the differentiation and identification of these two regioisomers by MS/MS.

Both fraction F10, containing Compound **1**, and a solution of Compound **2** (Figure 4) were also analyzed using NMR Spectroscopy. NMR spectral data (Supplementary information, Table N2, Figure N8, and 9) also confirmed the exact structures of regioisomeric Compound **1** and **2** (Figure 4). NMR and NOESY spectra confirmed the position of the methoxy and hydroxy group in compound **1** (Supplementary information, Figures N8 and 10).

### 3.3 Quantitative analysis

An external calibration curve was generated using measured amounts of the authentic standard of Compound **2**. The LLOD and LLOQ (described above, see Section 2.4) determined. The LLOD and LLOQ were estimated as 1.26 µg mL^–1^ and 4.22 µg mL^–1^ respectively, based on the signal to noise ratio. Linear response over the concentration range (5‐100 µg mL^–1^) was demonstrated (Supplementary information, Figure N2) which produced an equation for the line y = 128507x‐107121. The R^2^ value of the calibration curve was 0.991. The correlation coefficient (*r*) for this calibration curve was 0.995. For the purposes of quantitation, it was assumed that the capability to form ions under electrospray conditions was similar for the two isomers as they shared the same elemental composition and significant structural similarities.^29^ The amount of Compound **1** in the fraction F10 was quantitatively determined from the UPLC peak area using the standard calibration curve obtained from 5,7,3′‐trihydroxy‐4′‐methoxyflavone. Results suggested that *C. didymus* contained approximately 1.60 mg of Compound **1** per 10 g plant material.

## 4 CONCLUSIONS

High‐resolution UHPLC‐MS/MS, with tandem mass spectrometric analysis, via collision‐induced dissociation, was used to differentiate, identify and structurally characterize two regioisomeric methylated flavones by comparison and interpretation of their mass spectral fragmentation patterns from the aerial parts of the plant *C. didymus* used as an ethnomedicinal resource in parts of Asia. Theoretically separation of the methylated flavone regioisomers could be implemented by chiral chromatography but this can require significant cost time and may not be applicable in all cases. The ability to analyze these compounds by standard UHPLC‐MS/MS provides a more straightforward and practically accessible method with broad application. The product ion spectra of the two regioisomeric Compounds (**1** and **2**) were shown to have reproducibly different product ion abundances and these were used to differentiate the regio‐isomeric methoxylated flavonoids with identical chemical formulae and hence molecular masses. A possible fragmentation mechanism was suggested, accounting for the product ions observed from CID. This was based on the accurate mass values of the product ions coupled with information from the scientific literature. However, the mechanism remains speculative as no isotope labeling or modeling studies were pursued here to help assign the structure suggested. This study demonstrates the successful separation of methylated regioisomers using a rapid RP‐UHPLC‐MS/MS method providing potential for rapid identification and differentiation of regioisomeric plant natural products.

## AUTHOR CONTRIBUTIONS

Hafiza Noreen, Muhammad Farman, and James S.O. McCullagh conceptualized and designed the study. The following authors contributed to the acquisition of data: Hafiza Noreen, Muhammad Farman, and James S.O. McCullagh; data analysis: Hafiza Noreen, Muhammad Farman, James S.O. McCullagh, and Tim D.W. Claridge. Hafiza Noreen wrote the manuscript draft and Muhammad Farman, Tim D.W. Claridge, Edward Smith and James S.O. McCullagh edited the draft. All authors approved the submitted version of the article.

## CONFLICT OF INTEREST

The authors have declared no conflict of interest.

## Supporting information

Supplementary information

## Data Availability

Data are available on request from the authors.

## References

[1] Buckingham J , Munasinghe VRN . Dictionary of Flavonoids. 1st ed. Boca Raton, FL: CRC Press; 2015.

[2] Berim A , Hyatt DC , Gang DR . A set of regioselective *O*‐methyltransferases gives rise to the complex pattern of methoxylated flavones in sweet basil. Plant Physiol. 2012;160:1052‐1069. 22923679 10.1104/pp.112.204164 PMC3461529

[3] Kim B‐G , Jung B‐R , Lee Y , Hur H‐G , Lim Y , Ahn J‐H . Regiospecific flavonoid 7‐*O*‐methylation with *Streptomyces avermitilis O*‐methyltransferase expressed in *Escherichia coli* . J Agric Food Chem. 2006;54:823‐828. 16448189 10.1021/jf0522715

[4] Hsu YL , Kuo PL . Diosmetin induces human osteoblastic differentiation through the protein kinase C/p38 and extracellular signal‐regulated kinase 1/2 pathway. J Bone Miner Res. 2008;23:949‐960. 18269307 10.1359/jbmr.080219

[5] Lee SJ , Jung TH , Kim H , et al. Inhibition of c‐Kit signaling by diosmetin isolated from *Chrysanthemum morifolium* . Arch Pharm Res. 2014;37:175‐185. 23709168 10.1007/s12272-013-0158-7 PMC3906526

[6] Liao W , Ning Z , Chen L , et al. Intracellular antioxidant detoxifying effects of diosmetin on 2,2‐azobis(2‐amidinopropane) dihydrochloride (AAPH)‐induced oxidative stress through inhibition of reactive oxygen species generation. J Agric Food Chem. 2014;62:8648‐8654. 25075433 10.1021/jf502359x

[7] Yu G , Wan R , Yin G , et al. Diosmetin ameliorates the severity of cerulein‐induced acute pancreatitis in mice by inhibiting the activation of the nuclear factor‐κB. Int J Clin Exp Pathol. 2014;7:2133‐2142. 24966921 PMC4069971

[8] Androutsopoulos VP , Spandidos DA . The flavonoids diosmetin and luteolin exert synergistic cytostatic effects in human hepatoma HepG2 cells via CYP1A‐catalyzed metabolism, activation of JNK and ERK and P53/P21 up‐regulation. J Nutr Biochem. 2013;24:496‐504. 22749133 10.1016/j.jnutbio.2012.01.012

[9] Choi D‐Y , Lee JY , Kim M‐R , Woo E‐R , Kim YG , Kang KW . Chrysoeriol potentially inhibits the induction of nitric oxide synthase by blocking AP‐1 activation. J Biomed Sci. 2005;12:949‐959. 16228289 10.1007/s11373-005-9028-8

[10] Takemura H , Nagayoshi H , Matsuda T , et al. Inhibitory effects of chrysoeriol on DNA adduct formation with benzo[a]pyrene in MCF‐7 breast cancer cells. Toxicology. 2010;274:42‐48. 20553787 10.1016/j.tox.2010.05.009

[11] Khan AU , Gilani AH . Selective bronchodilatory effect of Rooibos tea (*Aspalathus linearis*) and its flavonoid, chrysoeriol. Eur J Nutr. 2006;45:463‐469. 17080260 10.1007/s00394-006-0620-0

[12] Liu Z , Song XD , Xin Y , et al. Protective effect of chrysoeriol against doxorubicin‐induced cardiotoxicity in vitro. Chin Med J (Engl). 2009;122:2652‐2656. 19951587

[13] Yang Y , Zhou X , Xiao M , et al. Discovery of chrysoeriol, a PI3K‐AKT‐mTOR pathway inhibitor with potent antitumor activity against human multiple myeloma cells in vitro. J Huazhong Univ Sci Technolog Med Sci. 2010;30:734‐740. 21181363 10.1007/s11596-010-0649-4

[14] Cha BY , Shi WL , Yonezawa T , Teruya T , Naga K , Woo JT . An inhibitory effect of chrysoeriol on platelet‐derived growth factor (PDGF)‐induced proliferation and PDGF receptor signaling in human aortic smooth muscle cells. J Pharmacol Sci. 2009;110:105‐110. 19423953 10.1254/jphs.08282fp

[15] Kim YH , Lee YS , Choi EM . Chrysoeriol isolated from *Eurya cilliata* leaves protects MC3T3‐E1 cells against hydrogen peroxide‐induced inhibition of osteoblastic differentiation. J Appl Toxicol. 2010;30:666‐673. 20981859 10.1002/jat.1539

[16] Noreen H , Farman M , McCullagh JSO . Bioassay‐guided isolation of cytotoxic flavonoids from aerial parts of *Coronopus didymus* . J Ethnopharmacol. 2016;194:971‐980. 27989879 10.1016/j.jep.2016.10.074

[17] Noreen H , Semmar N , Farman M , McCullagh JSO . Measurement of total phenolic content and antioxidant activity of aerial parts of medicinal plant *Coronopus didymus* . Asian Pac J Trop Med. 2017;10:792‐801. 28942828 10.1016/j.apjtm.2017.07.024

[18] Tomás‐Barberán FA , Ferreres F . Analytical methods of flavonols and flavones. In: Xu Z , Howard LR , eds. Analysis of Antioxidant‐Rich Phytochemicals. Hoboken, NJ: Wiley‐Blackwell; 2012:207‐246.

[19] Mabry TJ , Markham KR , Thomas MB . The ultraviolet spectra of flavones and flavonols. The Systematic Identification of Flavonoids. Berlin Heidelberg, NY: Springer‐Verlag; 1970:41‐164.

[20] Greenham J , Williams C , Harborne JB . Identification of lipophilic flavonols by a combination of chromatographic and spectral techniques. Phytochem Anal. 1995;6:211‐217.

[21] Greenham J , Harborne JB , Williams CA . Identification of lipophilic flavones and flavonols by comparative HPLC, TLC and UV spectral analysis. Phytochem Anal. 2003;14:100‐118. 12693635 10.1002/pca.693

[22] Stefova M , Stafilov T , Kulevanova S , Stefkov G , Bankova VS . QSRR of flavones: Evaluation of substituent contributions to RP HPLC retention. J Liq Chromatogr Relat Technol. 2007;30:1035‐1049.

[23] Li C , Schmidt A , Pichersky E , Shi F , Jones AD . Identification of methylated flavonoid regioisomeric metabolites using enzymatic semisynthesis and liquid chromatography‐tandem mass spectrometry. Metabolomics. 2013;9:92‐101.

[24] Justesen U . Collision‐induced fragmentation of deprotonated methoxylated flavonoids, obtained by electrospray ionization mass spectrometry. J Mass Spectrom. 2001;36:169‐178. 11288199 10.1002/jms.118

[25] Ma YL , Li QM , Van den Heuvel H , Claeys M . Characterization of flavone and flavonol aglycones by collision‐induced dissociation tandem mass spectrometry. Rapid Commun Mass Spectrom. 1997;11:1357‐1364.

[26] Ablajan K , Abliz Z , Shang X‐Y , He J‐M , Zhang R‐P , Shi J‐G . Structural characterization of flavonol 3,7‐di‐*O*‐glycosides and determination of the glycosylation position by using negative ion electrospray ionization tandem mass spectrometry. J Mass Spectrom. 2006;41:352‐360. 16432803 10.1002/jms.995

[27] Ferreres F , Gil‐Izquierdo A , Andrade PB , Valentão P , Tomás‐Barberán FA . Characterization of *C*‐glycosyl flavones *O*‐glycosylated by liquid chromatography‐tandem mass spectrometry. J Chromatogr A. 2007;1161:214‐223. 17602695 10.1016/j.chroma.2007.05.103

[28] Zhang X , Liu J , Xiong J , et al. Differentiation of isomeric methoxychalcones by electrospray ionization tandem mass spectrometry. Int J Mass Spectrom. 2018;434:100‐107.

[29] Vijlder TD , Valkenborg D , Lemière F , Romijn EP , Laukens K , Cuyckens F . A tutorial in small molecule identification via electrospray ionization‐mass spectrometry: The practical art of structural elucidation. Mass Spectrom Rev. 2018;37:607‐629. 29120505 10.1002/mas.21551 PMC6099382

