# Supplementary material for: Isolation, separation, identification, and quantification of bioactive methylated flavone regioisomers by UHPLC‐MS/MS
Source: Anal Sci Adv. 2021 May 5;2(7-8):364–72. doi: 10.1002/ansa.202100016 (PMC10989521; doi:10.1002/ansa.202100016)
Supplement: Supplementary file 1 — Supplementary information [file ANSA-2-364-s001.docx]

**Fig. N1.** The diagnostic MS/MS pattern for the deprotonated molecule [M-H]^-^ at *m/z* 299 of compound **1** at (**a**) 20, (**b**) 30 and (**c**) 40 eV collision energy.

**Fig. N2.** Calibration curve for 5,7,3'-trihydroxy-4'-methoxyflavone (standard).

**Fig. N3.** TIC profiles of compound **1** at five different concentrations. Intensities are relative to the peak obtained at 100 *µ*g mL^-1^.

**Fig. N4.** TIC profiles of compound **2** at five different concentrations. Intensities are relative to the peak obtained at 100 *µ*g mL^-1^.

**Fig. N5.** Mass spectra of compound **1** at five different concentrations.

**Fig. N6.** Mass spectra of compound **2** at five different concentrations.

**Fig. N7.** The diagnostic MS/MS pattern for the deprotonated molecule [M-H]^-^ at *m/z* 299 of (**a**) compound **1** and (**b**) compound **2** in negative ionization mode.

**Table N1**

UPLC-MS/MS spectroscopic data of Compound **1** and **2**.

| **S. No.** | **t_R_**  **(min.)** | **UV (nm)** | **MW** | **Molecular formula** | **MS**  **[M-H]^-^**  **Precursor ion (*m/z*)** | **MS/MS**  **Product ion peaks *m/z***  **(Relative abundance %)** | **Compound name** |
| --- | --- | --- | --- | --- | --- | --- | --- |
| Compound **1** | 5.02 | 224inf., 248, 251, 268, 291sh., 348 | 300 | C_16_H_12_O_6_ | 299 | 284.1743 (100), 256.1845 (36.16), 227.1854 (7.49), 151.1565 (8.50), 107.1619 (6.12) | 5,7,4'-Trihydroxy-3'-methoxyflavone |
| Compound **2** | 5.04 | 216inf., 222, 252, 268, 290sh., 346 | 300 | C_16_H_12_O_6_ | 299 | 284.1743 (100), 256.1845 (9.97), 227.1854 (7.50), 151.1565 (8.80), 107.1619 (7.45) | 5,7,3'-Trihydroxy-4'-methoxyflavone |

**Table N2**

^1^H-NMR spectroscopic data of compound **1** and **2**.

| Position | **1** | | **2** | | | | | |  |
| --- | --- | --- | --- | --- | --- | --- | --- | --- | --- |
|  | *δ*_H (ppm)_ | *J* (Hz) | | *δ*_H (ppm)_ | *J* (Hz) | |  |  |  |
| 3 | 6.661, 1H, s | - | | 6.613, 1H, s | | - | |  | |
| 6 | 6.239, 1H, d | ^4^*J* =2.1 | | 6.237, 1H, d | | ^4^*J* =2.1 | |  | |
| 8 | 6.498, 1H, d | ^4^*J* =2.1 | | 6.489, 1H, d | | ^4^*J* =2.1 | |  | |
| 2' | 7.516, 1H, d | ^4^*J* =2 | | 7.423, 1H, d | | ^4^*J* =2.1 | |  | |
| 3'-OCH_3_ | 3.990, 3H, s | - | | - | | - | |  | |
| 4'-OCH_3_ | - | - | | 3.992, 3H, s | | - | |  | |
| 5' | 6.965, 1H, d | ^3^*J* =8.5 | | 7.128, 1H, d | | ^3^*J* =8.4 | |  | |
| 6' | 7.544, 1H, dd | ^3^*J* =8.5, ^4^*J* =2 | | 7.540, 1H, dd | | ^3^*J* =7.0, ^4^*J* =2.1 | |  | |
| Spectra were obtained at 500MHz in MeOH-d_4_. | | | | | | | | |  |

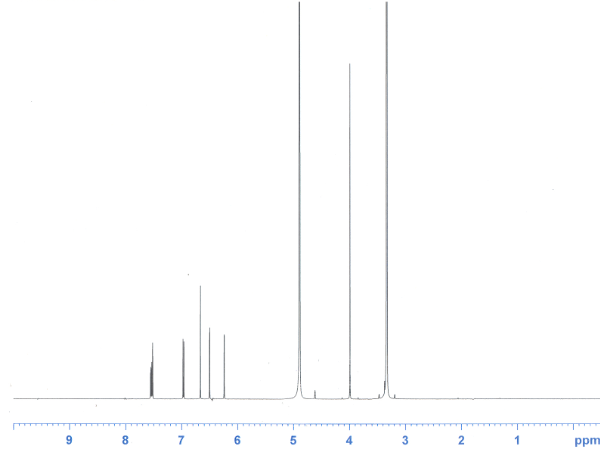


3

8

6

3’

2’

6’

5’

**Fig. N8**. ^1^H-NMR spectrum of compound **1**.


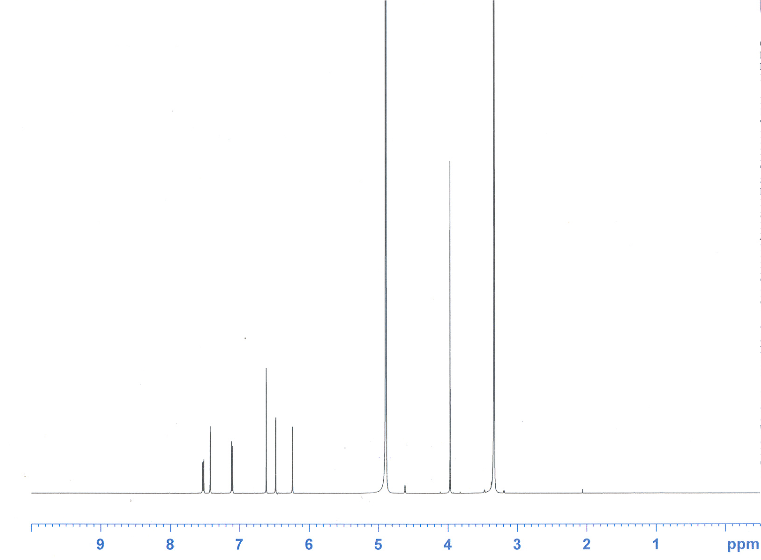


3

2’

6’

3’

5’

8

6

**Fig. N9**. ^1^H-NMR spectrum of compound **2**.


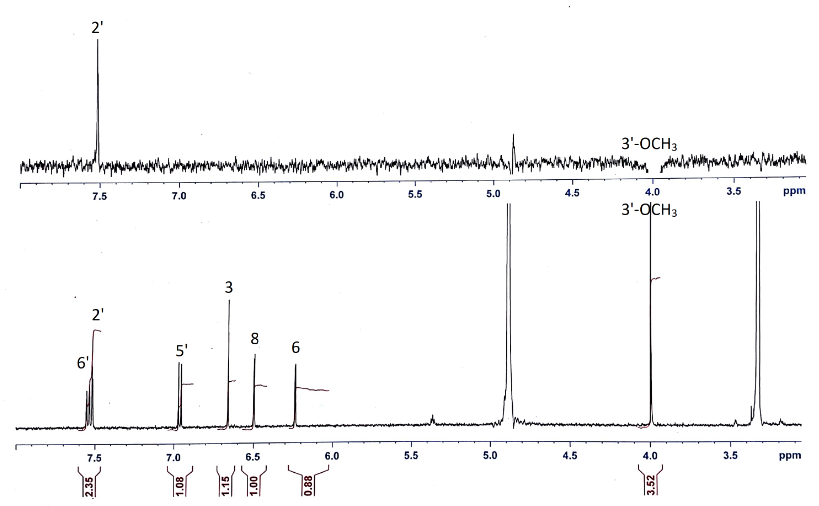


**Fig. N10**. ^1^H-NMR and 1-D NOESY spectra of compound **1**.

(A Bruker AVII 500 MHz NMR spectrometer was used for ^1^H-NMR. 1-D NOESY spectra were acquired using selective refocussing with a shaped gradient pulse (‘selnogp’ Bruker pulse sequence). The mixing time was 800 msec and selective excitation used an 80 msec 180° Gaussian shaped pulse).
